# A mast cell-associated apoptosis-related transcriptomic signature in clear cell renal cell carcinoma and *in vitro* characterization of YBX3

**DOI:** 10.3389/fimmu.2026.1894244

**Published:** 2026-09-03

**Authors:** Rijian Guan, Lijun Wan, Chunyun Feng

**Affiliations:** The Quzhou Affiliated Hospital of Wenzhou Medical University, Quzhou People’s Hospital, Quzhou, China

**Keywords:** apoptosis, clear cell renal cell carcinoma, innate immunity, mast cells, tumor microenvironment, YBX3

## Abstract

**Background:**

Clear cell renal cell carcinoma (ccRCC) exhibits substantial molecular and immune heterogeneity. Mast cells participate in tumor-microenvironment remodeling, but the prognostic relevance of mast cell-associated apoptosis-related transcriptional features remains unclear.

**Methods:**

Single-cell RNA sequencing was used to identify mast cells in ccRCC. Genes associated with the annotated mast-cell cluster were intersected with the HALLMARK_APOPTOSIS gene set to develop a mast cell-associated apoptosis score (MCAS) using machine-learning survival algorithms. MCAS was evaluated in TCGA-KIRC and two external cohorts. Associations with clinicopathological features, mutation profiles and model-derived genes were analyzed. YBX3 was functionally characterized using qRT-PCR, western blotting, CCK-8, colony formation, EdU, Transwell migration and flow cytometry assays.

**Results:**

A TPSAB1/CPA3-positive mast cell population was identified. The CoxBoost plus random survival forest model showed the best performance for MCAS construction. High MCAS scores predicted poor survival in the training and validation cohorts and were associated with advanced clinicopathological features and tumor mutation burden. SurvSHAP and SurvLIME jointly identified BNIP3, CX3CR1, HMOX1, and YBX3. High YBX3 expression was associated with unfavorable prognosis. Functionally, YBX3 knockdown inhibited proliferation, colony formation, DNA synthesis and migration, and promoted apoptosis with increased BAX and decreased Bcl-2, whereas YBX3 overexpression enhanced proliferative and migratory phenotypes.

**Conclusions:**

MCAS is a mast cell-associated apoptosis-related transcriptomic signature for prognostic stratification in ccRCC. The *in vitro* findings suggest that YBX3 may contribute to malignant phenotypes and apoptosis resistance in ccRCC cells.

## Introduction

Clear cell renal cell carcinoma (ccRCC) is the predominant histological subtype of renal cell carcinoma and is characterized by substantial molecular, cellular, and clinical heterogeneity (1, 2). Although advances in surgery, targeted therapy, and immune checkpoint blockade have improved patient outcomes, recurrence and distant metastasis remain common clinical challenges (3). Conventional clinicopathological factors, including tumor stage and nuclear grade, provide important prognostic information but do not fully explain the variation in outcomes among patients with otherwise similar disease characteristics. Molecular features reflecting both tumor-intrinsic biology and the tumor microenvironment may therefore provide complementary information for prognostic risk characterization.

The immune microenvironment is particularly relevant to ccRCC, which frequently exhibits extensive but functionally heterogeneous immune-cell infiltration (4, 5). Mast cells are tissue-resident innate immune cells that release cytokines, chemokines, growth factors, histamine, and proteases and can thereby influence angiogenesis, immune-cell recruitment, extracellular-matrix remodeling, and tumor-cell behavior (6). Their effects are context dependent and may be tumor-promoting or tumor-restraining according to their activation state, spatial localization, and interactions with malignant, immune, and stromal cells. This functional heterogeneity is reflected in ccRCC studies reporting divergent prognostic associations. Greater tumor-infiltrating mast-cell density has been associated with longer cancer-specific and relapse-free survival in nonmetastatic ccRCC (7) and with improved overall and progression-free survival in metastatic renal cell carcinoma (8). In contrast, increased mast-cell density has also been associated with higher tumor grade, advanced pathological stage, and metastasis (9). More recent single-cell and spatial transcriptomic analyses similarly showed that, although the overall mast-cell signature was associated with favorable prognosis, a VEGFA-positive mast-cell subset displayed potentially proangiogenic characteristics (10). These findings suggest that the clinical relevance of mast cells in ccRCC may depend more on their functional state and molecular context than on their overall abundance alone.

Several studies have provided potential biological explanations for the involvement of mast cells in ccRCC. Histopathological analyses demonstrated an association between mast-cell infiltration and microvessel density in renal cell carcinoma (11), while experimental studies implicated recruited mast cells in RCC angiogenesis and metastatic phenotypes through PI3K-AKT-GSK3β-adrenomedullin signaling (12). Mast cells may also contribute to an immunosuppressive microenvironment. In HIF-2α-high ccRCC, stem cell factor-mediated mast-cell recruitment was associated with immune evasion involving IL-10 and TGF-β (13). In PBRM1-mutated ccRCC, increased CCL5 expression was associated with mast-cell recruitment, reduced CD4-positive and CD8-positive T-cell infiltration, and enhanced angiogenic and immunosuppressive signaling (14). Bevacizumab-treated ccRCC specimens have also shown lower mast-cell and microvascular densities than untreated specimens, suggesting that mast-cell-related features may change in association with antiangiogenic treatment, although this observation does not establish a predictive biomarker of treatment response (15).

Apoptosis evasion is another important feature of ccRCC, enabling malignant cells to survive under hypoxic, inflammatory, immune, and therapeutic stress. Numerous apoptosis-related prognostic signatures have therefore been proposed for ccRCC (16). Mast-cell-associated inflammatory mediators and proteases provide a possible biological context in which immune signaling and apoptosis-related transcriptional states may intersect. For example, mast-cell-derived TNF-α can modulate BCL-2-associated apoptosis in endothelial cells under experimental conditions (17), whereas activation of protease-activated receptor 2 has been shown to reduce epithelial-cell apoptosis through MEK1/2- and PI3K-related pathways (18). These findings suggest that mast cells and apoptosis are not independent biological events; they may be associated with each other through cytokine release, protease activity, and downstream survival pathways.

Numerous immune-related and apoptosis-related prognostic signatures have been reported in ccRCC. The present study adopted a focused feature-derivation strategy by intersecting genes differentially expressed in a single-cell-defined mast-cell cluster with the human HALLMARK_APOPTOSIS gene set from the Hallmark collection of MSigDB. The resulting mast cell-associated apoptosis score (MCAS) was developed as an exploratory transcriptomic prognostic signature and evaluated across multiple ccRCC cohorts. Model-interpretation analyses identified candidate genes associated with MCAS, among which YBX3 was prioritized for *in vitro* characterization based on its model contribution, adverse prognostic association, tumor-associated expression pattern, and previously reported relevance to ccRCC. Here, “mast cell-associated” refers only to the computational origin of the candidate genes and does not indicate that MCAS directly measures mast-cell abundance or activity. Accordingly, this study investigated prognostic associations and YBX3-related cellular phenotypes rather than establishing a causal mast cell-YBX3-apoptosis pathway or predicting treatment response.

## Methods

### Data collection and preprocessing

The transcriptome data, clinical information, survival data and mutation data for ccRCC were obtained from public databases. The Cancer Genome Atlas Kidney Renal Clear Cell Carcinoma cohort (TCGA-KIRC) was used as the training group, while the GSE167573 (19) and E-MTAB-1980 datasets were used as the validation groups. Single-cell transcriptomic information was obtained from the Tumor Immune Single-cell Hub 2 (TISCH2) database using the curated dataset KIRC_GSE145281_aPDL1, which was derived from GSE145281 (20, 21). This TISCH2 dataset contains 44,220 cells from four patients with metastatic renal cell carcinoma in an anti-PD-L1 immunotherapy setting. Patient data without complete survival information were excluded. Before analysis, the gene expression data were standardized. For the external groups, their gene symbols were matched with those of the TCGA-KIRC dataset.

### Database-based single-cell transcriptomic analysis

Database-based single-cell analysis was performed using the uniformly processed KIRC_GSE145281_aPDL1 dataset in TISCH2. Cell identities and cluster assignments were obtained from the major-lineage and cluster-level annotations provided by TISCH2. The mast-cell population was identified from the TISCH2 major-lineage annotation and was primarily represented by cluster 22, designated Mast_C22. Its identity was supported by the expression of established mast-cell markers, including TPSAB1 and CPA3. Differentially expressed genes associated with Mast_C22 were obtained from the TISCH2 differential-expression results. Apoptosis-related transcriptional activity was examined using the HALLMARK_APOPTOSIS gene set. TISCH2-derived LISA transcription-factor enrichment and predicted cell-cell interaction results were used to characterize potential regulatory features and intercellular communication involving Mast_C22.

### Identification of mast cell-associated apoptosis-related genes

Apoptosis-related genes were obtained from the human HALLMARK_APOPTOSIS gene set (systematic name: M5902; H: Hallmark collection) in the Molecular Signatures Database (MSigDB, version 2026.1.Hs) (22). This gene set comprises genes involved in programmed cell death through caspase activation. Mast-cell-associated differentially expressed genes were intersected with the HALLMARK_APOPTOSIS genes. The overlapping genes were defined as mast cell-associated apoptosis-related genes and used as candidate features for model construction.

### Construction of MCAS

We compared various machine learning models to construct a prognostic prediction model based on the genes related to mast cell apoptosis. These models included CoxBoost, Random Survival Forest, Lasso, StepCox, Survival Support Vector Machine, GBM, and models related to ridge regression. The performance of these models was evaluated using the C-index in the TCGA-KIRC, GSE167573, and E-MTAB-1980 datasets. The model combining CoxBoost and Random Survival Forest showed the best overall performance. The key genes were selected through Random Survival Forest. The final score was named MCAS. Patients were divided into high- and low- MCAS groups using the median MCAS value calculated separately within each cohort. Kaplan-Meier (KM) analysis and time-dependent ROC curves were used to evaluate the prognostic effect.

### Mutation analysis

Somatic mutation data of TCGA-KIRC were analyzed using the maftools software package. The types of mutations, SNV classification, mutation frequency, and the most significant mutated genes were summarized. The tumor mutation burden was calculated and compared among different groups. Patients were stratified based on MCAS and TMB to evaluate their combined prognostic value. Meanwhile, the cancer-related pathway mutation patterns of the low MCAS group and the high MCAS group were compared.

### Model interpretation

In this study, two methods (SurvSHAP and SurvLIME) were employed to explain the MCAS model. SurvSHAP was used to assess the importance of global features and time-dependent effects, while SurvLIME was used to evaluate the contribution of local features. The genes identified through these two methods were selected as key genes. Subsequently, further analyses were conducted on the expression levels, diagnostic value, and prognostic significance of the genes BNIP3, CX3CR1, HMOX1, and YBX3. Candidate genes shared by SurvSHAP and SurvLIME were further prioritized for experimental validation according to their differential expression between ccRCC and normal tissues, diagnostic performance, direction and consistency of their associations with overall survival (OS), disease-specific survival (DSS), and progression-free interval (PFI), concordance with the adverse prognostic direction of MCAS, and previously reported biological relevance to ccRCC. The selected candidate was subsequently subjected to gain- and loss-of-function experiments.

### Cell culture and transfection

The cell lines used in this study were obtained from the Shanghai Cell Bank, Chinese Academy of Sciences. HK-2, OS-RC-2, 769-P, 786-O and Caki-1 cells were all cultured in RPMI-1640 medium containing 10% fetal bovine serum (FBS) at 37 °C and 5% CO_2_. In 769-P and 786-O cells, the expression of YBX3 was inhibited using siRNA technology. The experimental groups were siControl, siYBX3#1 and siYBX3#2. In Caki-1 cells, YBX3 was overexpressed using a YBX3 expression plasmid. The experimental groups were the vector group and the oe-YBX3 group.

### Cell viability assay

Cell viability was evaluated using the Cell Counting Kit-8 assay. Transfected cells were seeded into 96-well plates at a density of 5000 cells per well in 100 μL of complete culture medium. At 0, 24, 48, and 72 h after seeding, 10 μL of CCK-8 reagent was added to each well, and the cells were incubated for 2 h at 37 °C. Absorbance at 450 nm was measured using a microplate reader. Blank wells containing culture medium and CCK-8 reagent without cells were used for background correction.

### Colony formation assay

Transfected cells were seeded into six-well plates at a density of 1000 cells per well and cultured for 14 days. The medium was replaced every 3 days. When visible colonies had formed, the cells were washed with phosphate-buffered saline, fixed with 4% paraformaldehyde for 20 min, and stained with crystal violet for 20 min. Colonies containing at least 50 cells were counted using ImageJ. The colony-forming ability was expressed as the colony number and normalized relative to the control group.

### EdU assay

DNA synthesis was evaluated using an EdU incorporation assay kit according to the manufacturer’s instructions. Transfected cells were seeded into 6-well plates at a density of 2×10^5^ cells per well and incubated with 20 μM EdU for 2 h at 37 °C. The cells were subsequently fixed with 4% paraformaldehyde, permeabilized with 0.5% Triton X-100, and subjected to the EdU detection reaction. Cell nuclei were counterstained with DAPI. EdU-positive and total DAPI-positive cells were counted in at least five randomly selected fields per sample. The EdU-positive rate was calculated as the number of EdU-positive cells divided by the total number of DAPI-positive cells ×100%.

### Cell migration assays

Cell migration was evaluated using uncoated Transwell inserts with an 8-μm pore size. Briefly, 20,000 transfected cells suspended in 200 µL serum-free medium were seeded into the upper chamber, while 600 µL medium containing 10% fetal bovine serum was added to the lower chamber as a chemoattractant. After incubation for 24 h, cells remaining on the upper surface of the membrane were gently removed with a cotton swab. Cells that had migrated to the lower surface were fixed with 4% paraformaldehyde, stained with crystal violet, photographed, and counted in randomly selected microscopic fields.

### Apoptosis assay

After transfection, 769-P and 786-O cells were collected and stained with Annexin V-FITC and propidium iodide according to the manufacturer’s instructions. The stained cells were analyzed by flow cytometry. The total apoptotic fraction was calculated as the sum of early apoptotic cells (Annexin V-FITC-positive/PI-negative) and late apoptotic cells (Annexin V-FITC-positive/PI-positive).

### Database-derived assessment of YBX3 protein and spatial expression

Publicly available immunohistochemical images of YBX3 in normal kidney and ccRCC tissues were retrieved from the Human Protein Atlas (HPA) database. The representative images and corresponding HPA annotations were examined descriptively to provide supportive evidence for the tissue-level protein expression pattern of YBX3. Spatial transcriptomic data were obtained from the SpatialTME portal, an integrated database for the visualization and analysis of spatial tumor-microenvironment data (23). In the present study, one spatial transcriptomic section, GSM5420752 (patient 24; n = 1), was examined. The surgically resected specimen was embedded in optimal cutting temperature compound and flash-frozen. A 10-μm section was placed on a 10x Genomics Visium Gene Expression slide, methanol-fixed, stained with hematoxylin and eosin, and imaged at 10× magnification. Sequencing was performed using the Illumina NovaSeq 6000 platform. The original sequencing data were processed using Space Ranger v1.2.0 and aligned to the GRCh38 human reference genome. Spot-level YBX3 expression and the deconvolution-based predicted spatial distribution of tumor cells were examined using the Spatial Gene Expression and Spatial Cell Pattern modules of SpatialTME. Standard Visium spots are 55 μm in diameter and spaced 100 μm apart from center to center; therefore, each spot may contain transcripts from multiple cells and does not represent single-cell resolution. The spatial analysis was used descriptively as database-derived supportive evidence, and no independent spatial cohort comparison or statistical validation was performed.

### Statistical analysis

Statistical analysis was conducted using R software and GraphPad Prism software. For comparisons of continuous variables, the Student’s t-test, Wilcoxon rank sum test, analysis of variance (ANOVA), or Kruskal-Wallis test was employed depending on the specific circumstances. Survival analysis was performed using the KM curve and the log-rank test. The hazard ratio and its 95% confidence interval were calculated using Cox regression. A two-sided P value less than 0.05 was considered statistically significant.

## Results

### Single-cell characterization of mast cells in ccRCC

**Figure 1:**
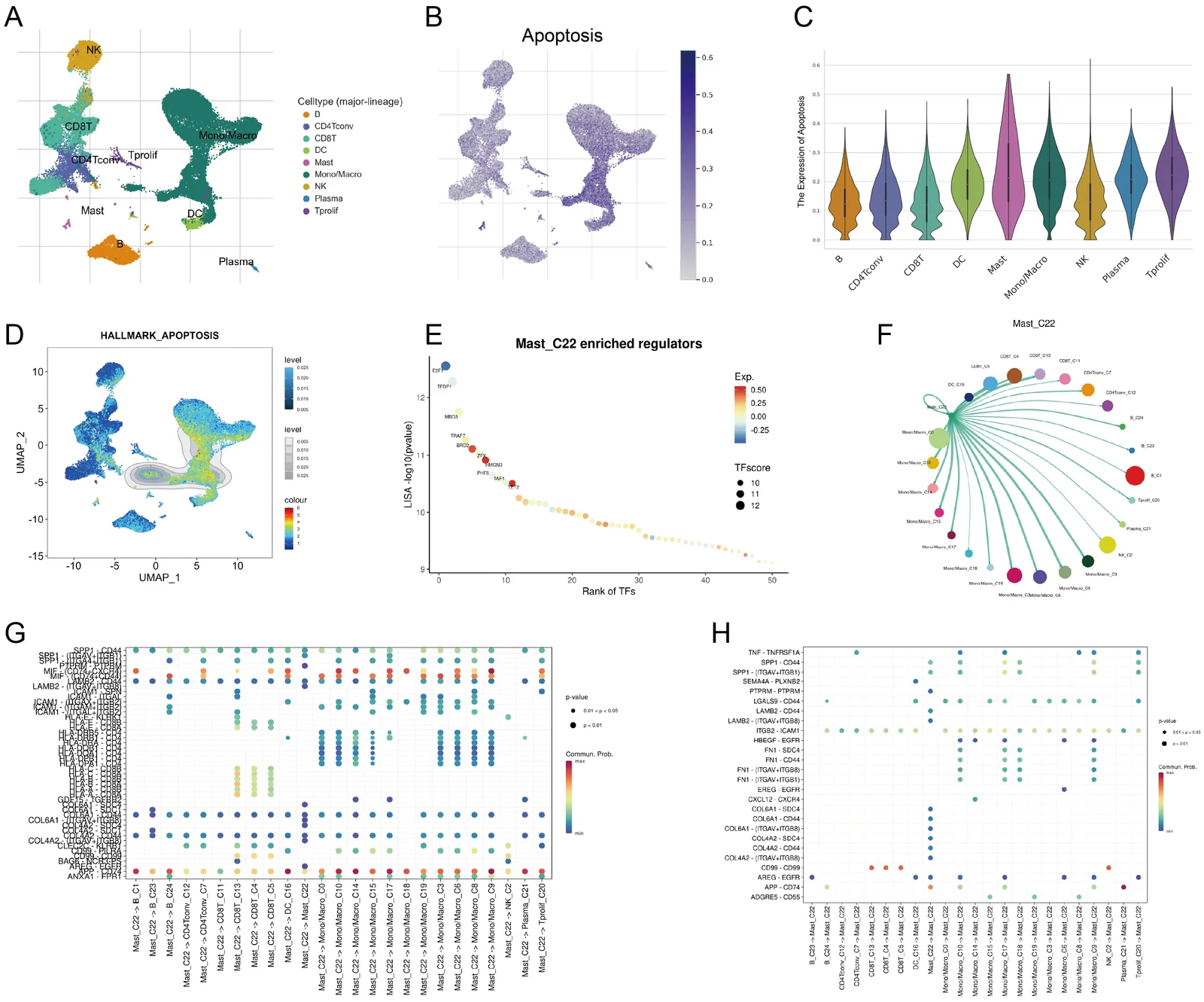
Single-cell characterization of mast cells in ccRCC. **(A)** UMAP of nine major immune-cell lineages in GSE145281. **(B)** UMAP distribution of apoptosis-related activity scores. **(C)** Apoptosis-related scores across immune-cell lineages. **(D)** UMAP visualization of HALLMARK_APOPTOSIS activity. **(E)** LISA-ranked candidate regulators enriched in Mast_C22; dot color and size represent relative expression and transcription-factor score, respectively. **(F)** Predicted communication network between Mast_C22 and other immune-cell subclusters. **(G, H)** Predicted outgoing and incoming ligand-receptor interactions of Mast_C22, respectively. Dot color indicates communication probability, and dot size indicates statistical significance.

Single-cell analysis of GSE145281 identified nine major immune-cell lineages, including a distinct mast-cell population (**Figure 1A**). Apoptosis-related activity varied across immune-cell types, with mast cells showing a heterogeneous distribution of apoptosis scores (**Figures 1B–D**). Further analysis identified Mast_C22 as the mast-cell cluster and suggested several candidate regulators, including E2F1, TFDP1, MBD3, BRD2, ZFX, and TET2 (**Figure 1E**). Cell-cell communication analysis predicted extensive interactions between Mast_C22 and other immune-cell subsets (**Figure 1F**), involving outgoing signals such as SPP1, MIF, ICAM1, and HLA-related molecules and incoming signals such as TNF-TNFRSF1A, SPP1-CD44, LGALS9-CD44, and CXCL12-CXCR4 (**Figures 1G, H**). These findings characterize Mast_C22 as a transcriptionally distinct population associated with apoptosis-related activity and predicted immune-cell communication, without establishing direct regulation of tumor-cell apoptosis.

### Machine learning constructs a prognostic model based on mast cell-associated apoptosis-related genes in ccRCC

**Figure 2:**
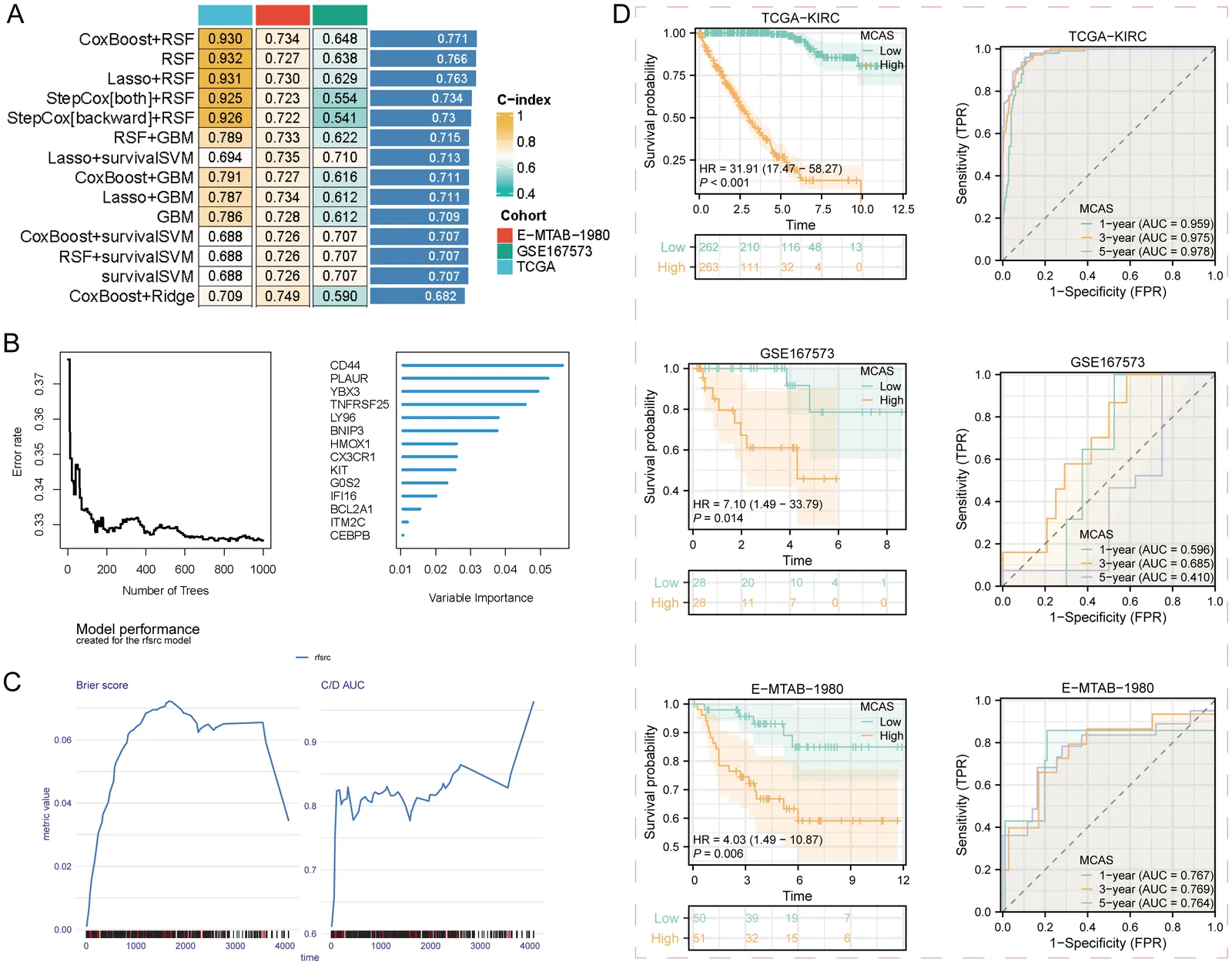
Development and cross-cohort assessment of MCAS. **(A)** Comparison of C-index values for different machine learning models based on mast cell-associated apoptosis-related genes across TCGA-KIRC, GSE167573, and E-MTAB-1980 cohorts. **(B)** Random survival forest model showing error rate and variable importance of candidate genes. **(C)** Model performance evaluation based on the Brier score and time-dependent cumulative/dynamic AUC. **(D)** Kaplan-Meier (KM) survival curves and time-dependent ROC curves of MCAS in TCGA-KIRC, GSE167573, and E-MTAB-1980 cohorts.

Differentially expressed genes associated with Mast_C22 were obtained from the TISCH2 differential-expression results and intersected with the HALLMARK_APOPTOSIS gene set. The overlapping genes were used as candidate features for prognostic model construction. This step restricted model construction to genes that were both associated with the mast-cell cluster and included in the apoptosis-related gene set. We compared various machine learning models in three cohorts (TCGA-KIRC, GSE167573, and E-MTAB-1980). Among them, the CoxBoost + RSF model showed the best overall performance, with an average C-index of 0.771 (**Figure 2A**). Subsequently, the random survival forest model was used to further screen the key genes. As the number of trees increased, the error rate decreased, indicating an improvement in model stability. Variable importance analysis determined the key genes in the model (**Figure 2B**). Model performance over time was further evaluated using the Brier score and time-dependent cumulative/dynamic area under the curve (AUC) (**Figure 2C**). Based on these genes, the MCAS was calculated, and patients were divided into high MCAS group and low MCAS group according to the median of MCAS. In the TCGA-KIRC study group, patients with high MCAS levels had significantly lower survival probabilities than those with low MCAS levels. In the external cohorts, high MCAS remained associated with poorer survival, but the time-dependent discriminatory performance was lower and varied between cohorts. In GSE167573, the 1-, 3-, and 5-year AUC values were 0.596, 0.685, and 0.410, respectively, indicating limited discrimination at several time points. In E-MTAB-1980, the corresponding AUC values were 0.767, 0.769, and 0.764, indicating moderate discrimination (**Figure 2D**). Collectively, MCAS showed prognostic stratification across the evaluated cohorts, although its external predictive performance was heterogeneous and weaker than that observed in the training cohort.

### Exploratory clinical evaluation of MCAS in ccRCC

**Figure 3:**
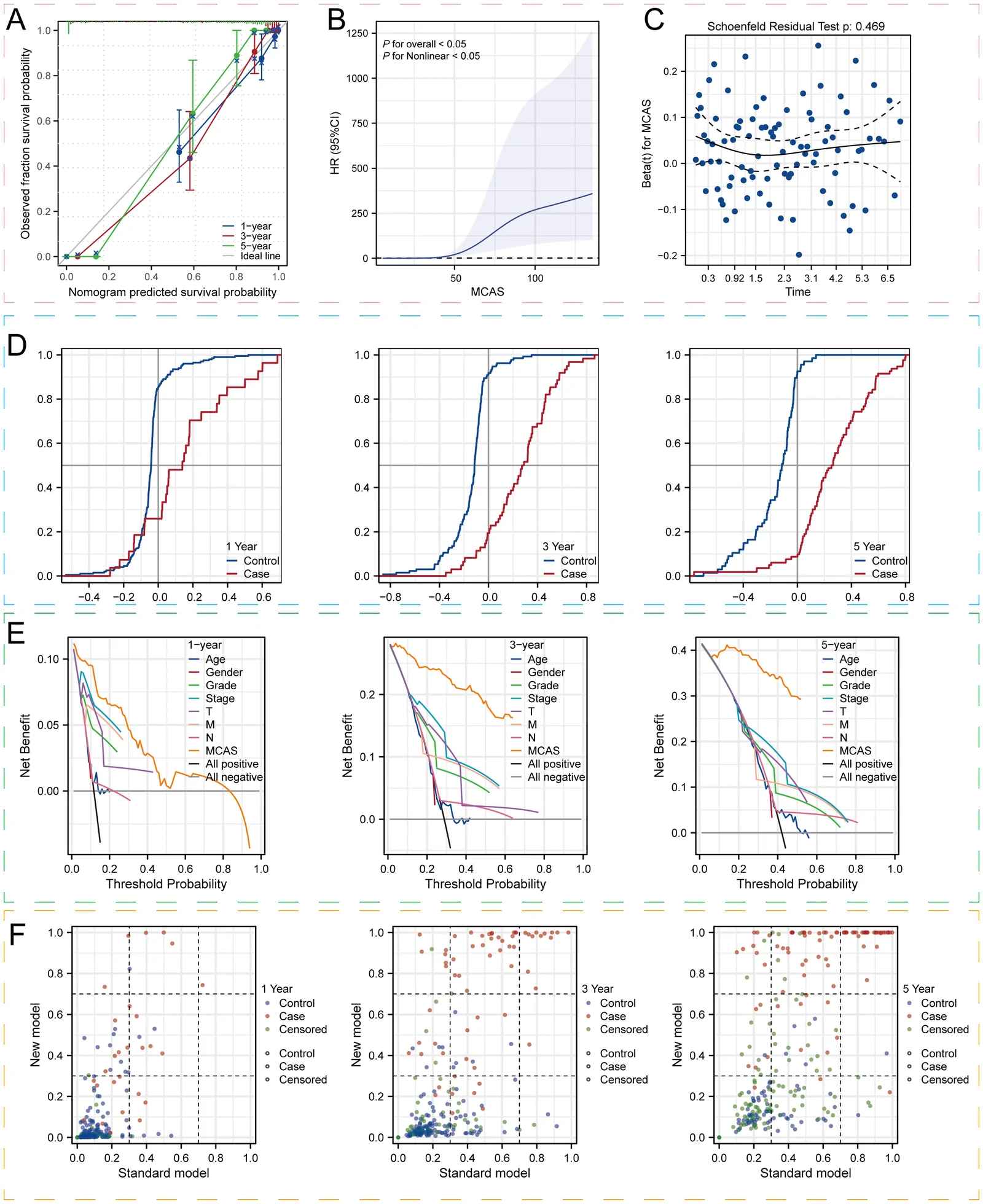
Clinical evaluation of MCAS-based prognostic prediction in ccRCC. **(A)** Calibration curves comparing model-predicted and observed survival probabilities at 1, 3, and 5 years. **(B)** Restricted cubic spline analysis showing the association between MCAS and hazard ratio. **(C)** Schoenfeld residual test evaluating the proportional hazards assumption for MCAS. **(D)** Risk distribution plots comparing predicted probabilities between cases and controls at 1, 3, and 5 years. **(E)** Decision curve analysis comparing the net benefit of MCAS with common clinical variables at 1, 3, and 5 years. **(F)** Reclassification plots comparing the standard model and the new model after adding MCAS at 1, 3, and 5 years.

We further performed an exploratory clinical evaluation of MCAS in TCGA-KIRC. The calibration curves showed apparent agreement between model-predicted and observed survival probabilities at 1, 3, and 5 years (**Figure 3A**). Restricted cubic spline analysis suggested a nonlinear association between MCAS and mortality risk (**Figure 3B**). The Schoenfeld residual test did not indicate a significant violation of the proportional hazards assumption (P = 0.469; **Figure 3C**). Predicted risks tended to be higher among cases than among controls, particularly at 3 and 5 years (**Figure 3D**). Exploratory decision-curve and reclassification analyses suggested possible incremental prognostic information from MCAS (**Figures 3E, F**).

### MCAS is associated with advanced clinicopathological features and poor survival across ccRCC subgroups

Next, we investigated the relationship between the MCAS score and the common clinical and pathological features of ccRCC. MCAS progressively increased with tumor grade: the MCAS level was the highest in patients with grade G4 tumors, and the lowest in patients with grade G1 tumors. The stacked bar plot showed that as the tumor grade increased, the proportion of the high MCAS group also increased, and there was a significant difference between the G1 and G4 groups. Subsequently, survival analysis was conducted for different grade subgroups: in patients with grades 1–2 and 3-4, the survival probability of the high MCAS group was lower than that of the low MCAS group (**Supplementary Figure 1A**). The clinical stage also showed a similar trend: the MCAS score continued to rise from stage I to stage IV, and the proportion of high MCAS was higher in advanced patients (**Supplementary Figure 1B**). In terms of T stage, the MCAS level was the highest in patients with T4 tumors (**Supplementary Figure 1C**), and the proportion of high MCAS increased with the progression of T stage. Patients with distant metastasis also had higher MCAS scores. Compared with M0 patients, the proportion of high MCAS cases was higher in M1 patients (**Supplementary Figure 1D**). Regarding the lymph node status, the MCAS score of N1 patients was higher than that of N0 patients, and the proportion of high MCAS cases in the N1 group also increased. The survival curve showed that in the N0 and N1 subgroups, high MCAS was associated with poorer survival (**Supplementary Figure 1E**).

### MCAS is associated with tumor mutation burden in ccRCC

**Figure 4:**
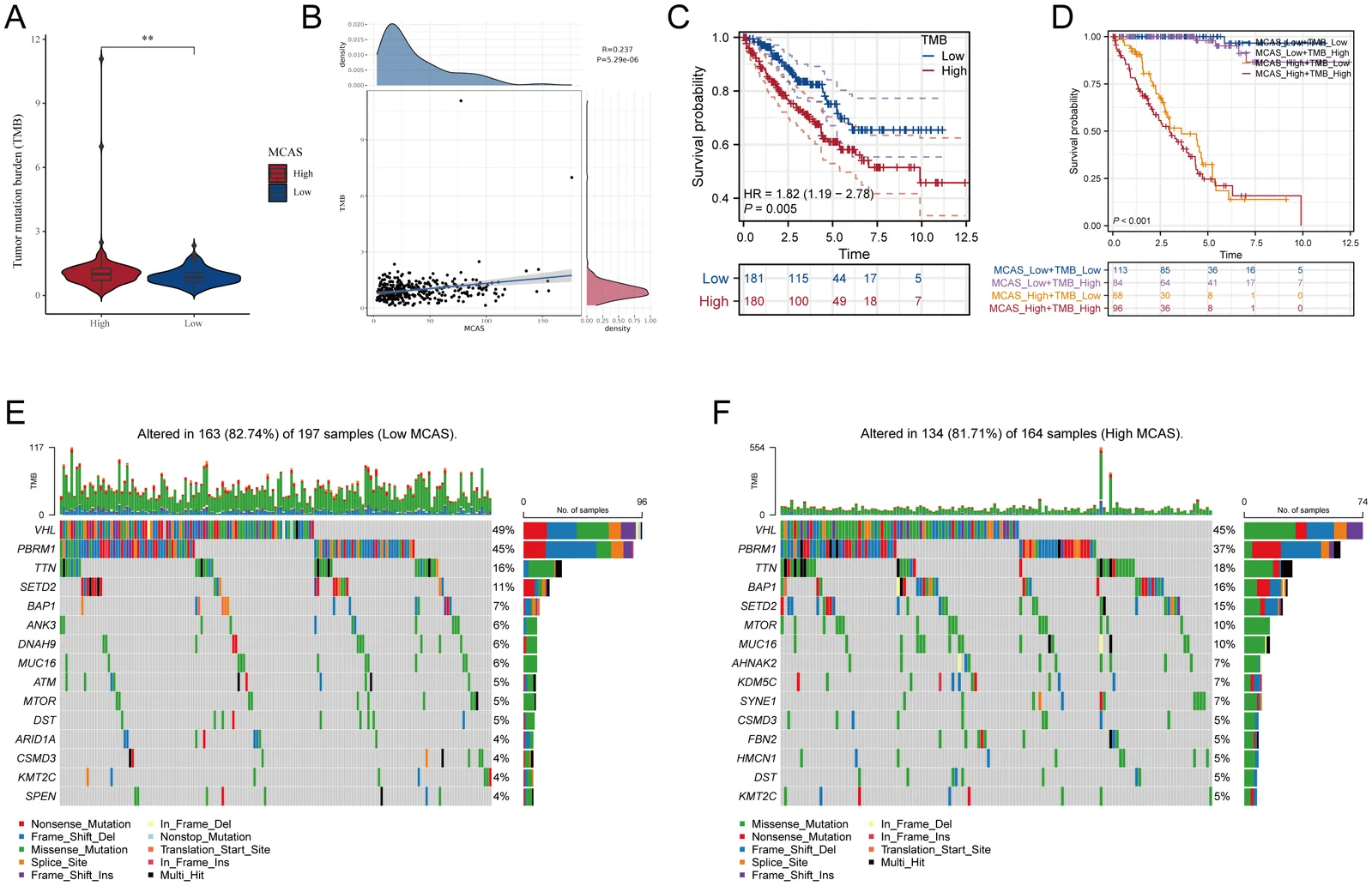
Comparison of TMB and somatic mutation characteristics between the MCAS groups in ccRCC. **(A)** Comparison of TMB between the high- and low-MCAS groups. **(B)** Correlation analysis between TMB and MCAS. **(C)** KM survival analysis comparing high- and low-TMB groups. **(D)** KM survival analysis based on combined MCAS and TMB stratification. **(E, F)** Mutation waterfall plots of the low- and high-MCAS groups.

Next, we analyzed the genomic characteristics related to MCAS in ccRCC patients. The mutation summary showed that missense mutations were the predominant variant classification, whereas single-nucleotide variants were the most common variant type (**Supplementary Figure 2A**). Among the six substitution classes, C>T was the most frequent, followed by C>A and T>C (**Supplementary Figure 2B**). TMB was significantly higher in the high MCAS group and was positively correlated with MCAS (**Figures 4A, B**). VHL and PBRM1 were the most frequently mutated genes in the cohort. Survival analysis showed that patients with higher TMB had a lower survival probability than those with lower TMB (**Figure 4C**). Combined MCAS–TMB stratification separated patients into four groups with significantly different overall survival (**Figure 4D**). Further comparison of the mutation characteristics showed that among the 197 low MCAS samples, 163 samples harbored detectable mutations, accounting for 82.74% of the total cases (**Figure 4E**). Among the 164 high MCAS samples, 134 samples harbored detectable mutations, accounting for 81.71% of the total cases (**Figure 4F**). The mutation analysis at the pathway level indicated that both MCAS groups had abnormalities in multiple cancer-related pathways. In the low MCAS group, the affected pathways included RTK-RAS, NOTCH, WNT, Hippo, PI3K, MYC, NRF2, TP53, TGF-β, and cell cycle pathways (**Supplementary Figure 2C**). Similar pathway patterns were also observed in the high MCAS group (**Supplementary Figure 2D**). The proportion of affected pathways in both groups for RTK-RAS and NOTCH pathways was relatively high.

### Model interpretation identifies key contributors to the MCAS prognostic model

**Figure 5:**
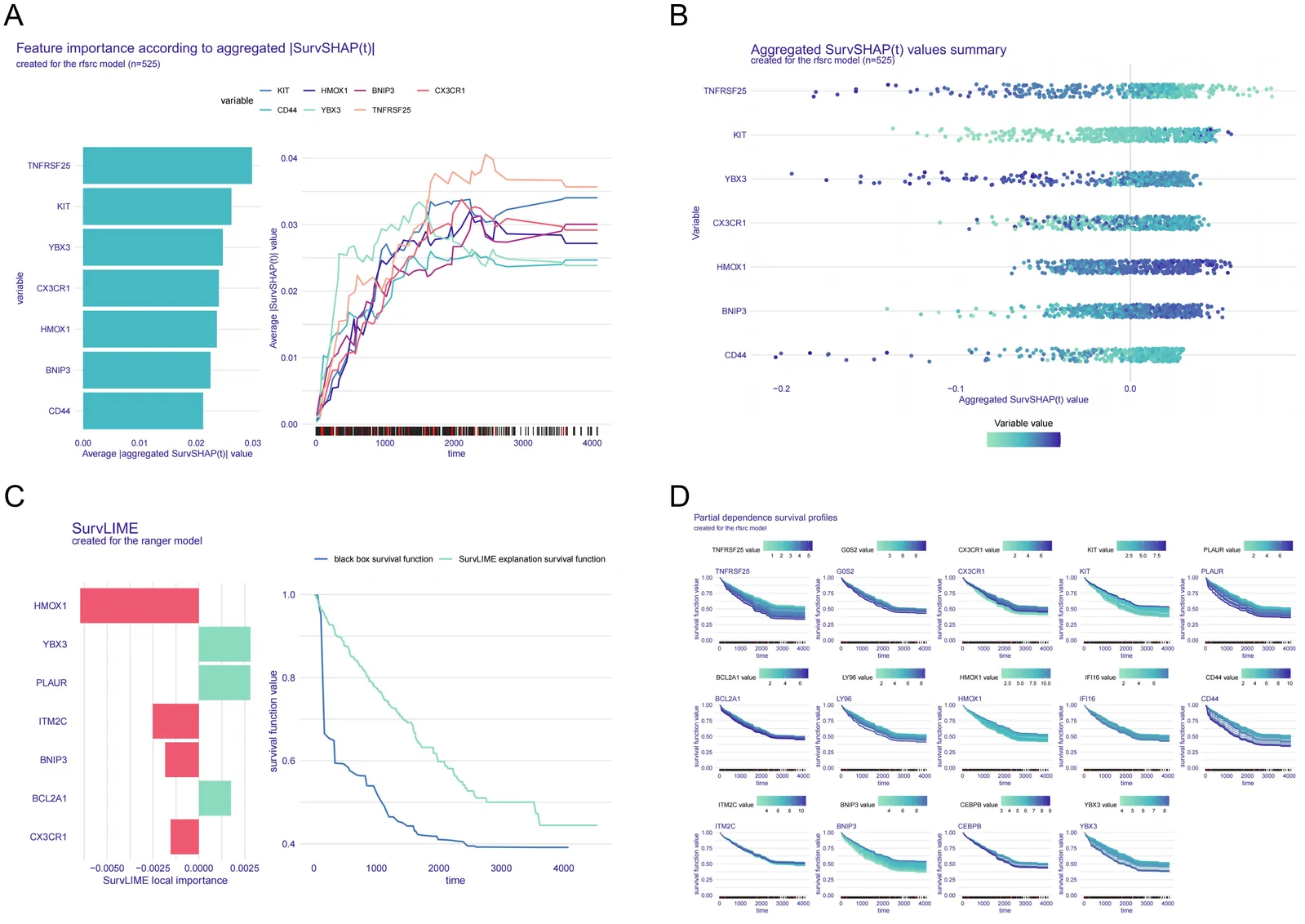
Interpretation of the MCAS prognostic model using SurvSHAP and SurvLIME. **(A)** Feature importance ranking and time-dependent SurvSHAP contribution curves for the ranger model. **(B)** Aggregated SurvSHAP value summary showing the contribution distribution of key genes. **(C)** SurvLIME analysis showing local feature importance and the SurvLIME-derived local surrogate survival function. **(D)** Partial dependence survival profiles of model genes in the MCAS prognostic model.

To gain a deeper understanding of how the MCAS model generates survival prediction results, we employed two tools, SurvSHAP and SurvLIME, to explain the model. The SurvSHAP analysis revealed that several genes, including TNFRSF25, KIT, YBX3, CX3CR1, HMOX1, BNIP3, and CD44, made relatively large contributions to the random survival forest model (**Figure 5A**). The aggregated SurvSHAP chart further revealed the direction and distribution characteristics of each gene’s contribution over time (**Figure 5B**). Different genes exhibited unique contribution patterns, indicating that the model is not dependent on a single biomarker but is jointly shaped by multiple genes related to apoptosis and immunity. The SurvLIME analysis identified HMOX1, YBX3, PLAUR, ITM2C, BNIP3, BCL2A1, and CX3CR1 as locally important variables in the ranger model (**Figure 5C**). The interpretation curves of SurvLIME clearly demonstrated the differences between the black-box survival function and the interpretable survival function, illustrating the local approximation used by this method. Through partial-dependence survival profile analysis, we clarified the specific impact of each model gene on the prediction of survival probability (**Figure 5D**). These results helped identify candidate genes that contributed substantially to MCAS-based survival predictions.

### Integrated model interpretation identifies four key MCAS-related genes and prioritizes YBX3 for experimental validation

**Figure 6:**
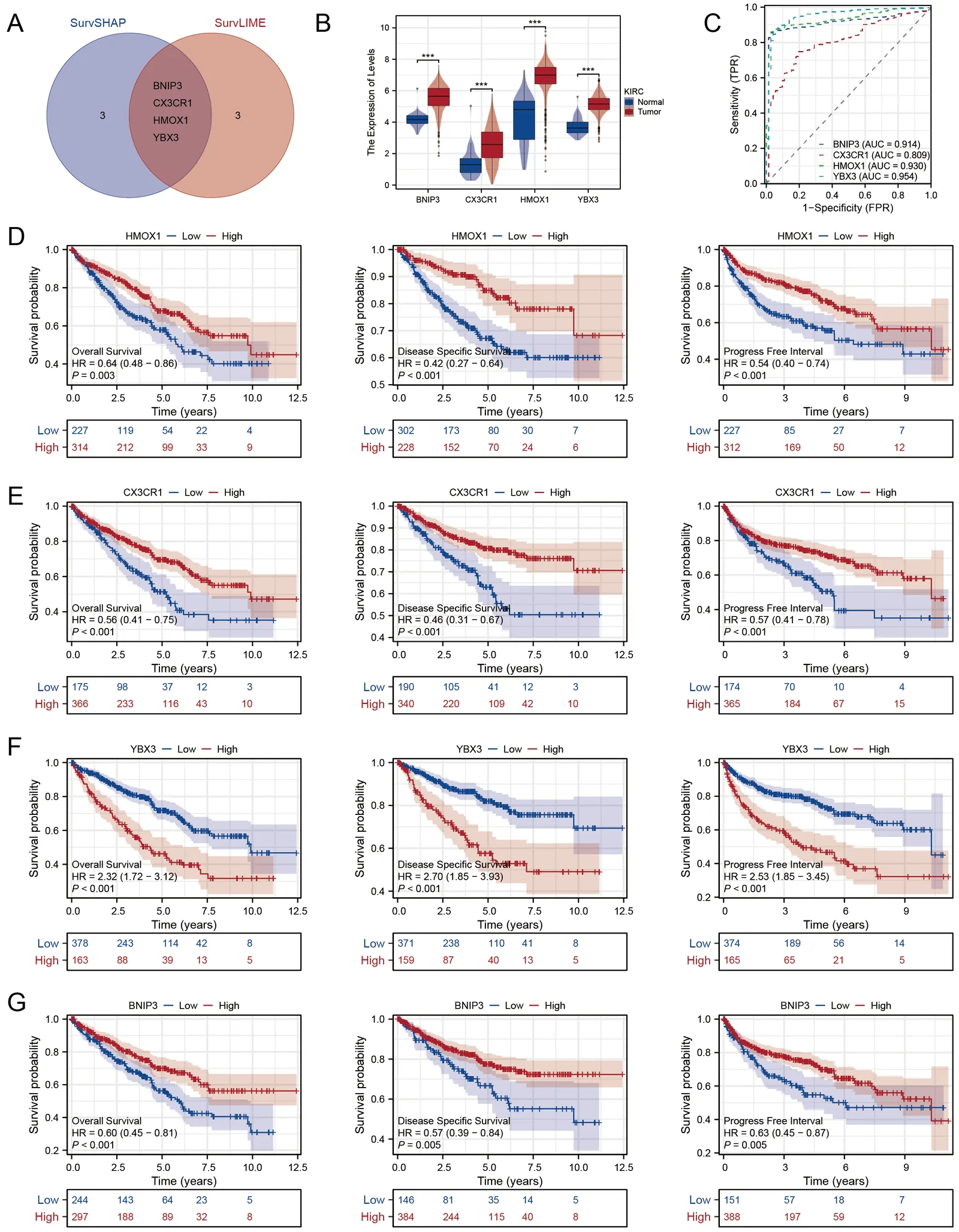
Identification and characterization of key MCAS-related genes in ccRCC. **(A)** Venn diagram showing the shared genes identified by SurvSHAP and SurvLIME. **(B)** Expression levels of BNIP3, CX3CR1, HMOX1, and YBX3 in normal and ccRCC tumor tissues. **(C)** ROC curves evaluating the diagnostic performance of BNIP3, CX3CR1, HMOX1, and YBX3. **(D–G)** KM survival analysis of HMOX1 **(D)**, CX3CR1 **(E)**, YBX3 **(F)**, and BNIP3 **(G)** for overall survival, disease-specific survival, and progression-free interval.

The intersection of the SurvSHAP and SurvLIME results identified four consistently prioritized model-associated genes: BNIP3, CX3CR1, HMOX1, and YBX3 (**Figure 6A**). All four genes were differentially expressed between ccRCC and normal kidney tissues (**Figure 6B**) and showed varying abilities to distinguish tumor from normal samples. Among them, YBX3 had the highest diagnostic AUC (0.954), compared with HMOX1 (0.930), BNIP3 (0.914), and CX3CR1 (0.809) (**Figure 6C**). The four genes also differed in the direction of their prognostic associations. Higher expression of HMOX1, CX3CR1, and BNIP3 was associated with favorable survival outcomes, whereas higher YBX3 expression was consistently associated with unfavorable OS, DSS, and PFI (**Figures 6D–G**). More importantly, YBX3 was the only candidate whose high expression was consistently associated with unfavorable outcomes across all three survival endpoints, which was concordant with the adverse prognostic direction of high MCAS. By contrast, higher expression of BNIP3, CX3CR1, and HMOX1 was predominantly associated with favorable survival. YBX3 was also upregulated in ccRCC tissues, and previous evidence has implicated it as an oncogenic RNA-binding protein in ccRCC (24). Therefore, based on the convergence of model interpretability, diagnostic performance, survival direction, tumor-associated expression, and biological plausibility, YBX3 was prioritized for experimental validation.

### YBX3 knockdown suppresses proliferation and migration of ccRCC cells

**Figure 7:**
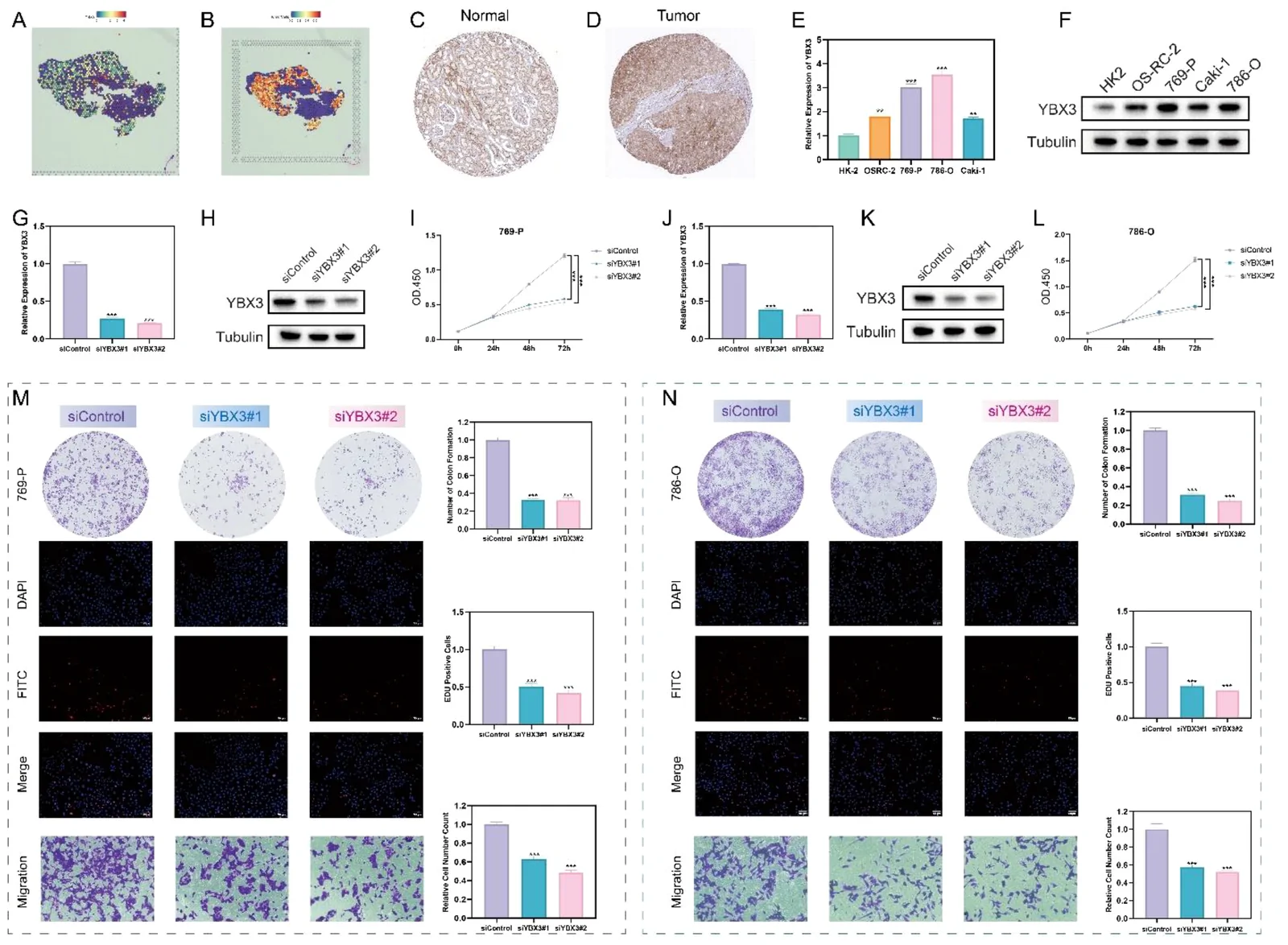
Database-derived assessment and experimental characterization of YBX3 in ccRCC. **(A)** Spot-level YBX3 expression in GSM5420752. **(B)** Deconvolution-based predicted spatial distribution of tumor cells in the same section. **(C, D)** Representative immunohistochemical staining of YBX3 in normal kidney and ccRCC tumor tissue. **(E)** Quantitative analysis of YBX3 expression in HK-2 and ccRCC cell lines. **(F)** Western blot analysis of YBX3 protein expression in HK-2 and ccRCC cell lines. **(G, J)** Quantitative analysis of YBX3 knockdown efficiency in 769-P and 786-O cells. **(H, K)** Western blot confirmation of YBX3 knockdown in 769-P and 786-O cells. **(I, L)** CCK-8 assay showing cell viability after YBX3 knockdown in 769-P and 786-O cells. **(M, N)** Colony formation, EdU staining, and migration assays after YBX3 knockdown in 769-P and 786-O cells.

To validate the biological role of YBX3 in ccRCC, we first examined its expression pattern in tumor and normal tissues. Spatial maps showed descriptive overlap between YBX3 expression and predicted tumor-cell-enriched regions (**Figures 7A, B**). Consistently, representative immunohistochemical images retrieved from the HPA database showed stronger YBX3 staining in ccRCC tissues than in normal kidney tissues (**Figures 7C, D**). We then detected YBX3 expression in ccRCC cell lines and normal renal epithelial cells. Compared with HK-2 cells, YBX3 expression was increased in OS-RC-2, 769-P, 786-O, and Caki-1 cells (**Figures 7E, F**). Among these cell lines, 769-P and 786-O showed relatively high YBX3 expression, so they were selected for knockdown experiments. Two siRNAs were used to silence YBX3 in 769-P and 786-O cells. Both siYBX3#1 and siYBX3#2 reduced YBX3 expression at the mRNA and protein levels, confirming effective knockdown (**Figures 7G, H**, J, K). CCK-8 assays showed that YBX3 knockdown reduced cell viability over time in both 769-P and 786-O cells (**Figures 7I, L**). Functional assays further showed that YBX3 silencing weakened the malignant phenotype of ccRCC cells. In 769-P cells, knockdown of YBX3 reduced colony formation, EdU-positive cells, and the number of migrated cells (**Figure 7M**). Similar changes were observed in 786-O cells (**Figure 7N**). These findings suggest that YBX3 may contribute to ccRCC cell growth and migration *in vitro*.

### YBX3 overexpression enhances proliferation and migration of ccRCC cells

**Figure 8:**
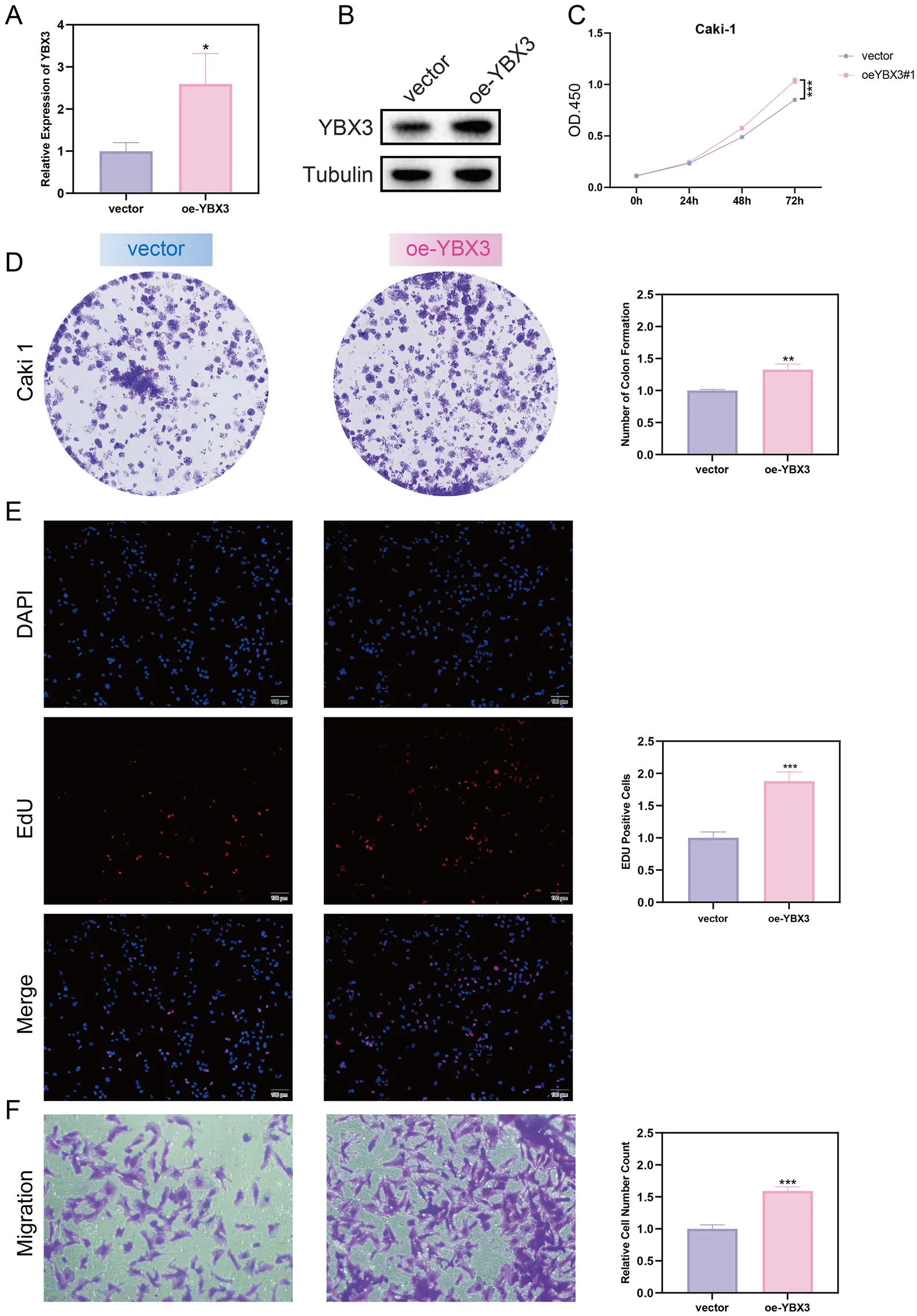
Effects of YBX3 overexpression on proliferative and migratory phenotypes in Caki-1 cells. **(A)** Quantitative analysis of YBX3 overexpression efficiency in Caki-1 cells. **(B)** Western blot confirmation of YBX3 overexpression in Caki-1 cells. **(C)** CCK-8 assay showing cell viability after YBX3 overexpression. **(D)** Colony formation assay showing the clonogenic ability of Caki-1 cells after YBX3 overexpression. **(E)** EdU staining showing proliferating cells after YBX3 overexpression. **(F)** Transwell migration assay showing the migration ability of Caki-1 cells after YBX3 overexpression.

To further confirm the role of YBX3 in ccRCC cells, we performed YBX3 overexpression experiments in Caki-1 cells. YBX3 overexpression was confirmed by qRT-PCR and western blotting. Compared with the vector group, the oe-YBX3 group showed higher YBX3 expression at both mRNA and protein levels (**Figures 8A, B**). The CCK-8 assay showed that YBX3 overexpression increased the viability of Caki-1 cells over time, especially at 72 h (**Figure 8C**). The colony formation assay also showed that cells with YBX3 overexpression formed more colonies than control cells (**Figure 8D**). EdU staining further supported this result. The oe-YBX3 group had more EdU-positive cells than the vector group, indicating enhanced DNA synthesis and cell proliferation (**Figure 8E**). Transwell migration assay showed that YBX3 overexpression increased the number of migrated Caki-1 cells (**Figure 8F**). These findings are consistent with the YBX3 knockdown results and suggest that YBX3 may contribute to ccRCC cell proliferation and migration *in vitro*.

### YBX3 knockdown is associated with increased apoptosis in ccRCC cells

**Figure 9:**
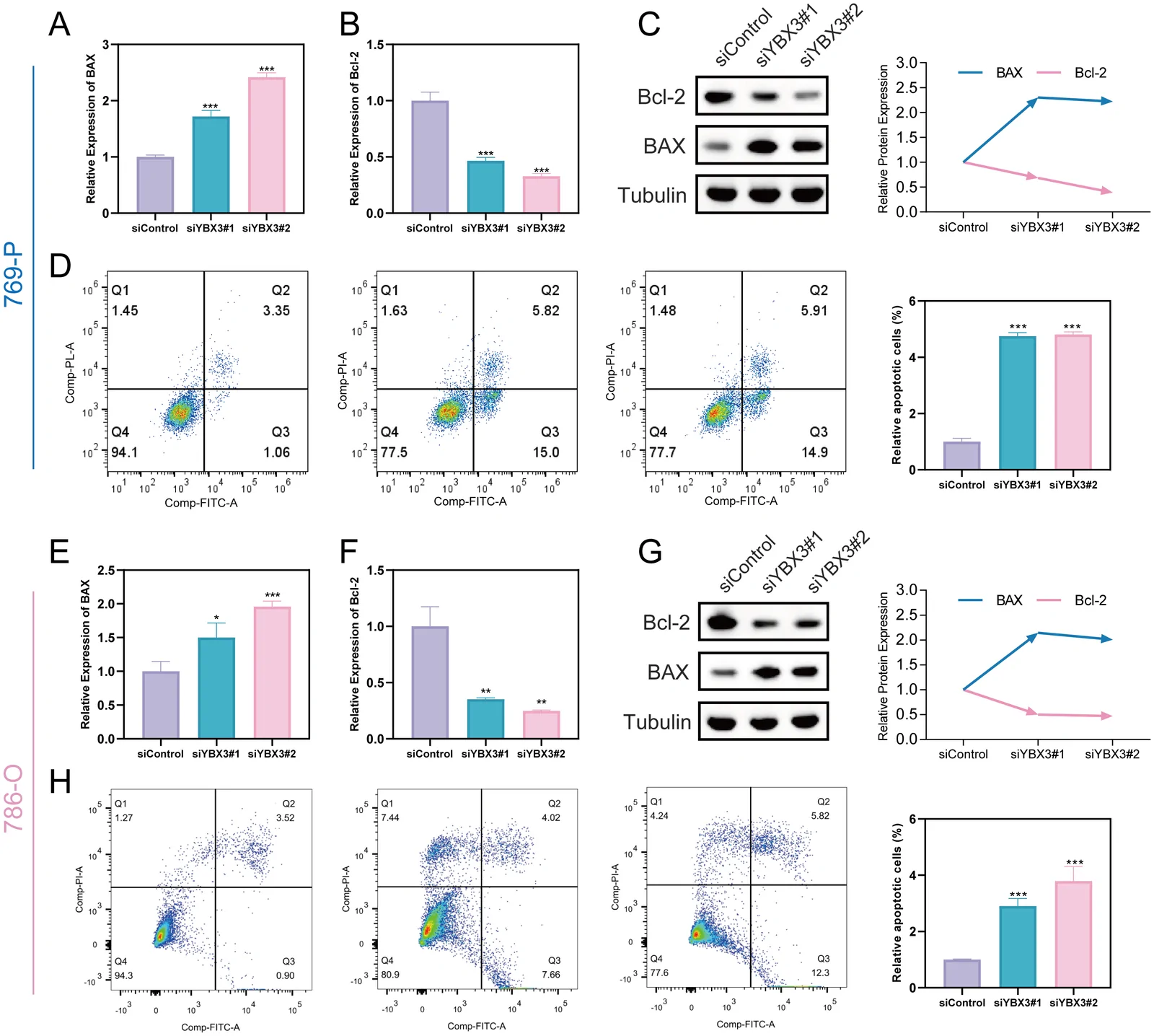
YBX3 knockdown is associated with increased apoptosis in ccRCC cells. **(A, B)** Relative mRNA expression of BAX and Bcl-2 after YBX3 knockdown in 769-P cells. **(C)** Western blot analysis of BAX and Bcl-2 protein expression in 769-P cells after YBX3 knockdown. **(D)** Flow cytometry analysis of apoptosis in 769-P cells after YBX3 knockdown. **(E, F)** Relative mRNA expression of BAX and Bcl-2 after YBX3 knockdown in 786-O cells. **(G)** Western blot analysis of BAX and Bcl-2 protein expression in 786-O cells after YBX3 knockdown. **(H)** Flow cytometry analysis of apoptosis in 786-O cells after YBX3 knockdown.

To determine whether YBX3 affects apoptosis in ccRCC cells, we detected apoptosis-related markers after YBX3 knockdown. In 769-P cells, silencing YBX3 increased BAX expression and decreased Bcl-2 expression at the mRNA level (**Figures 9A, B**). Western blot analysis showed the same trend at the protein level, with increased BAX and reduced Bcl-2 after YBX3 knockdown (**Figure 9C**). Flow cytometry was then used to evaluate apoptosis. Compared with the siControl group, both siYBX3#1 and siYBX3#2 increased the proportion of apoptotic 769-P cells (**Figure 9D**). This result suggests that loss of YBX3 weakens the anti-apoptotic state of 769-P cells. Similar results were observed in 786-O cells. YBX3 knockdown reduced Bcl-2 expression and increased BAX expression at the mRNA and protein levels (**Figures 9E–G**). Flow cytometry also showed a higher apoptotic cell proportion in the siYBX3 groups than in the siControl group (**Figure 9H**).

## Discussion

In this study, we developed MCAS as a transcriptomic prognostic signature by intersecting differentially expressed genes identified in a single-cell-defined mast-cell cluster with genes in the human HALLMARK_APOPTOSIS gene set from the Hallmark collection of MSigDB. MCAS was associated with survival and clinicopathological characteristics in ccRCC, although its discriminatory performance varied substantially across cohorts. SurvSHAP and SurvLIME jointly identified BNIP3, CX3CR1, HMOX1, and YBX3 as model-associated candidates. YBX3 was prioritized for functional investigation, and gain- and loss-of-function experiments showed that its expression was associated with proliferation, migration, and apoptosis-related phenotypes in ccRCC cells. These findings support the potential prognostic relevance of MCAS and suggest that YBX3 may contribute to malignant phenotypes *in vitro*, but they do not establish a coordinated mast-cell-driven apoptosis program or a causal mast cell-YBX3 pathway.

Mast cells are functionally heterogeneous components of the ccRCC microenvironment. Greater mast-cell density has been associated with favorable survival in some nonmetastatic and metastatic RCC cohorts (7, 8), whereas other studies have linked increased mast-cell infiltration to advanced pathological features and metastasis (9). In HIF-2α-high ccRCC, stem cell factor-mediated mast-cell recruitment has been associated with immune evasion involving IL-10 and TGF-β (13). In PBRM1-mutated ccRCC, CCL5-associated mast-cell recruitment was accompanied by reduced T-cell infiltration and enhanced angiogenic and immunosuppressive signaling. Recent single-cell analyses have also suggested that the overall mast-cell signature and specific states, such as a VEGFA-positive subset, may have different prognostic associations (10). Thus, the role of mast cells in ccRCC may depend on their activation state, spatial distribution, and molecular context rather than overall abundance alone.

In our single-cell analysis, a relatively small TPSAB1/CPA3-positive cluster was annotated as mast cells and designated Mast_C22. Although TPSAB1 and CPA3 are widely used mast-cell markers, annotation based mainly on these markers has limitations. Low-level expression or ambient RNA contamination may reduce the cellular specificity of marker-gene expression in droplet-based single-cell datasets (25). Additional markers such as KIT, MS4A2, HDC, and FCER1A, together with protein-level validation, would increase annotation confidence. The limited mast-cell number and use of a single scRNA-seq dataset also prevented robust characterization of multiple mast-cell states. Cell-cell communication analysis predicted interactions involving SPP1-CD44, MIF-associated signaling, ICAM1-related interactions, TNF-TNFRSF1A, LGALS9-CD44, CXCL12-CXCR4, and extracellular-matrix-associated ligand-receptor pairs. However, predicted communication networks can vary according to the analytical method and interaction database used (26), and transcriptomic co-expression does not demonstrate ligand secretion, receptor binding, downstream pathway activation, or functional effects on recipient cells.

Mast-cell-derived cytokines and proteases provide a possible context in which inflammatory signaling and apoptosis-related transcriptional states may intersect. For example, mast-cell-derived TNF-α can modulate BCL-2-associated endothelial-cell apoptosis (17), whereas PAR2 activation can reduce epithelial-cell apoptosis through MEK1/2- and PI3K-related signaling. Mast-cell-mediated immunosuppression could also indirectly reduce cytotoxic lymphocyte-mediated apoptotic pressure on tumor cells. More broadly, resistance to regulated cell death has been linked to immune-microenvironment remodeling in ccRCC. These observations provide biological plausibility for an intersection between immune signaling and apoptosis-related states but do not demonstrate that mast cells directly regulate apoptosis in ccRCC cells.

Transcriptomic signatures derived from immune-associated or regulated cell-death programs are already abundant in ccRCC. Previous studies have developed prognostic frameworks based on integrated programmed cell-death patterns and Treg-associated programmed cell death (27, 28). Therefore, the novelty of MCAS lies primarily in its focused mast-cell-associated feature-derivation strategy rather than in the general concept of a cell-death-related prognostic model. Moreover, “mast cell-associated” refers to the computational origin of the candidate genes. These genes are not necessarily exclusive to mast cells, and their expression in bulk tissue may arise from malignant, immune, or stromal compartments.

MCAS should be interpreted as an exploratory mast cell-associated apoptosis-related transcriptomic signature rather than a direct measure of mast-cell abundance or activity, because its designation reflects the computational origin of its candidate genes and their expression in bulk tissues may derive from multiple cellular compartments. Its performance and clinical relevance also require caution. The high 1-, 3-, and 5-year AUCs in TCGA-KIRC (0.959, 0.975, and 0.978) represent apparent performance and may be affected by overfitting, while discrimination was attenuated in GSE167573 (0.596, 0.685, and 0.410) and E-MTAB-1980 (0.767, 0.769, and 0.764). Because these additional cohorts contributed to algorithm selection, they were not completely independent validation cohorts, and repeated cross-validation and permutation testing were not performed. Cohort-specific median cutoffs were used for survival visualization and do not constitute a universal threshold. Moreover, incomplete information on histological necrosis, ECOG performance status, and other required variables prevented direct comparisons with the Leibovich, SSIGN, and UISS systems; therefore, the calibration, decision-curve, and reclassification analyses should be considered exploratory and do not establish clinical actionability. Similarly, although TMB was statistically higher in the high-MCAS group, the proportions of patients with detectable mutations were nearly identical between the low- and high-MCAS groups (82.74% and 81.71%), with broadly similar mutated genes and pathway patterns. Thus, neither marked differences in genomic instability nor superiority over established prognostic tools can be inferred, and MCAS requires rigorous independent validation before any clinical application.

YBX3 was prioritized because it was identified by both model-interpretation methods, showed the highest tumor-versus-normal AUC among the four candidates, and was the only candidate whose higher expression was consistently associated with unfavorable OS, DSS, and PFI. This direction was concordant with the adverse-risk pattern of high MCAS, and previous research has implicated YBX3 as a potential oncogenic RNA-binding protein in ccRCC (24). YBX3 has also been shown experimentally to bind and stabilize selected solute-carrier mRNAs, supporting its role in post-transcriptional regulation (29). Nevertheless, this selection does not demonstrate that YBX3 is the most biologically important MCAS gene or that it is regulated by mast cells. YBX3 knockdown reduced viability, colony formation, DNA synthesis, and migration in 769-P and 786-O cells, whereas its overexpression enhanced proliferative and migratory phenotypes in Caki-1 cells. YBX3 knockdown also increased apoptosis, accompanied by increased BAX and decreased BCL-2. These findings suggest that YBX3 may contribute to malignant and apoptosis-related phenotypes *in vitro*. However, they do not establish that BAX or BCL2 transcripts are direct YBX3 targets. RNA-binding, mRNA stability, translational, upstream signaling, and mitochondrial assays were not performed. Furthermore, no mast cell-ccRCC co-culture, conditioned-medium, or ligand-receptor perturbation experiment was conducted. The YBX3 experiments therefore represent functional characterization of a model-associated candidate rather than validation of a mast cell-YBX3-apoptosis axis.

Several additional limitations should be acknowledged. This retrospective study used public datasets with heterogeneous platforms, sample composition, follow-up, and clinical annotations. Treatment histories were incomplete, and treatment-related confounding cannot be excluded. No immunotherapy-treated cohort was analyzed, so MCAS cannot be interpreted as a predictor of immunotherapy response. The cross-sectional data did not permit assessment of MCAS stability during disease progression or treatment. Finally, MCAS was evaluated only in ccRCC, and its specificity to this tumor type remains unknown. Prospective, longitudinal, treatment-annotated studies using prespecified modeling pipelines, fully independent cohorts, and mechanistic mast cell-tumor experiments are required to establish the reproducibility, biological basis, and potential clinical relevance of MCAS.

## Conclusion

In summary, this study developed a mast cell-associated apoptosis-related transcriptomic signature with prognostic value in ccRCC. YBX3 was identified as a model-associated candidate, and the *in vitro* gain- and loss-of-function experiments suggest that YBX3 may contribute to malignant phenotypes and apoptosis resistance in ccRCC cells. Further *in vivo* and mechanistic studies are required to determine its role in ccRCC progression.

## Data Availability

The original contributions presented in the study are included in the article/**Supplementary Material**. Further inquiries can be directed to the corresponding author.

## Supplementary material

Supplementary Figure 1 Association between MCAS and clinicopathological features in ccRCC. **(A–E)** MCAS distribution, survival analysis, and MCAS proportion across different tumor grades **(A)**, clinical stages **(B)**, T stages **(C)**, distant metastasis status **(D)**, lymph node status **(E)**.

Supplementary Figure 2 Association of MCAS with tumor mutation burden in ccRCC. **(A)** Summary of variant classification, variant type, SNV class, variants per sample, variant-classification distribution, and the top 10 mutated genes. **(B)** Distribution of single-nucleotide substitution classes across samples. **(C, D)** Cancer-related pathway mutation profiles in the low- and high-MCAS groups, respectively.
